# Supplementary material for: Going “Green” in the Prevention and Management of Atherothrombotic Diseases: The Role of Dietary Polyphenols
Source: J Clin Med. 2021 Apr 3;10(7):1490. doi: 10.3390/jcm10071490 (PMC8038361; doi:10.3390/jcm10071490)
Supplement: Supplementary file 1 [file jcm-10-01490-s001.pdf]

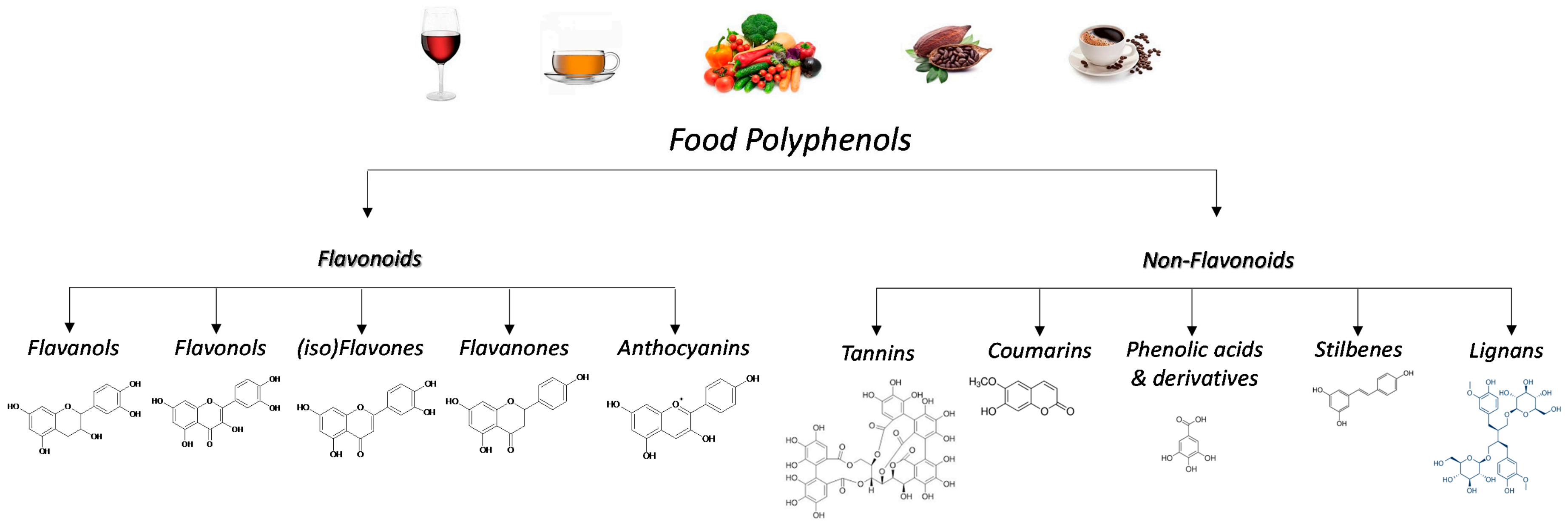

Supplementary Figure 1. Main classes of food polyphenols, and representative chemical structures. (For additional information on food sources please see Ref [7,10,14,16]).
